# UsCoTc: Improved Collaborative Filtering (CFL) recommendation methodology using user confidence, time context with impact factors for performance enhancement

**DOI:** 10.1371/journal.pone.0282904

**Published:** 2023-03-15

**Authors:** Mahesh T. R., V. Vinoth Kumar, Se-Jung Lim

**Affiliations:** 1 Department of Computer Science and Engineering, Faculty of Engineering and Technology, JAIN (Deemed-to-be University), Bangalore, India; 2 School of Information Technology and Engineering, VIT University, Vellore, Tamil Nadu, India; 3 Liberal Arts & Convergence Studies, Honam University, Gwangsan-gu, Gwangju-si, Republic of Korea; Jeonbuk National University, REPUBLIC OF KOREA

## Abstract

In today’s society, time is considered more valuable than money, and researchers often have limited time to find relevant papers for their research. Identifying and accessing essential information can be a challenge in this situation. To address this, the personalized suggestion system has been developed, which uses a user’s behavior data to suggest relevant items. The collaborative filtering strategy has been used to provide a user with the top research articles based on their queries and similarities with other users’ questions, thus saving time by avoiding time-consuming searches. However, when rating data is abundant but sparse, the usual method of determining user similarity is relatively straightforward. Furthermore, it fails to account for changes in users’ interests over time resulting in poor performance. This research proposes a new similarity measure approach that takes both user confidence and time context into account to increase user similarity computation. The experimental results show that the proposed technique works well with sparse data, and improves accuracy by 16.2% compared to existing models, especially during prediction. Furthermore, it enhances the quality of recommendations.

## Introduction

The exponential increase of digital information and density of data on social media can lead to conflicting information, hindering timely access to relevant information on the internet. Retrieval systems have partially solved this issue, but they lack features such as prioritization and customization. Recommender System (RS) is a type of data retrieval system that addresses the issue of information explosion. Massive Quantity of periodic data is generated when users express their choices, interests or behaviors regarding an item. Based on the user’s background, an RS can determine whether or not a specific user will favor a resource asset.

Various applications, such as search engines as well as recommender systems, utilize user models. Researchers with similar tastes in search results can aid in the discovery of an effective Recommender System (RS) by enabling more efficient search, however, sharing search outcomes is often too cumbersome and time-consuming to be practical. A research paper recommender system can help researchers find the best and most relevant research articles in their field based on ratings from other researchers with similar interests.

With the use of collaborative or content-based filtering (CBF), recommender systems are used to recommend products or items to interested individuals. In collaborative filtering (CFL) systems, the model is built using the user’s behavior and the choices of other users and is then used to anticipate what items would be of interest to the user. In Content-based filtering approach, items with identical features are recommended utilizing a sequence of discrete item characteristics. Hybrid Recommender Systems are created by combining these methodologies [1].

With the vast amount of information available on the internet, making the right decision is becoming increasingly challenging [2]. Personalized recommender systems (RS) have been widely used in e-commerce sites to provide user-specific information recommendations [3]. Examples include Jing Dong Mall and Amazon’s book suggestion service [4] which provide tailored recommendations for a variety of consumers. The key to personalized systems [5] is choosing the right recommendation algorithm. The goal of CFL suggestion is to generate a list of appealing products for consumers based on the tastes of their like-minded neighborhood. To create hybrid recommendations, these two methodologies are often combined [6].

CFL is one of the most widely used and successful technologies in the field of personalized recommendation systems. CFL algorithm was proposed first time in this [7]. The basic concept is that users’ previous preferred behaviors have a major influence on their future behavior, and that their past behavior is consistent and indicative of their future behavior [8]. This article [9] categorizes the CFL algorithm into two types: CFL recommendation based on memory (MeBCFL) and CFL recommendation based on model (MoBCFL). MeBCFL recommendation relies on extensive use of legacy data to find related products.

The MeBCFL suggestion can be further divided into MoBCFL recommendation and user-based CFL recommendation, each approached from a distinct standpoint. The MoBCFL recommendation creates a new model based on user features and rating information and then uses it to estimate the target user’s potential project rating. Furthermore, past studies have shown that the similarity measure has a significant impact on the recommendation algorithm’s prediction accuracy. The similarity measure is an important aspect of a CFL-based Recommender System. It is used to identify the collection of users, who will have similar preferences towards the chosen content. Traditional similarity metrics, such as the cosine-based similarity (CBS), Pearson-correlation coefficient (PECC), Euclidean distance-based similarity (EDBS), and the adjusted cosine-based similarity (ACBS) have often been used to evaluate similarity in CFLs [10].

In this research, we introduce a novel similarity measure called Collaborative-Filtering (CFL) recommendation method based on Time Context and User Confidence (User_UsCoTc), a new strategy that improvises on the ACBS, with the goal of improving standard CFL algorithms to reach a good amount of accuracy. User_UsCoTc takes into account not only the user’s trustworthiness compared to others, but also the dynamics of the user’s interests. The results of the experiments indicate that User_UsCoTc not only increases the similarity estimate, but also accurately discovers more neighbors and improves prediction accuracy.

### Motivation of the research

During the research investigation, personalized suggestions are given to the users in recommendation platforms using knowledge mining techniques. In order to effectively extract essential information, it is important to assess the data stored in the databases of the systems. However, the large volume of research data and number of researchers can pose a challenge for recommender systems.

CFL-based strategies use the analysis of user ratings to uncover correlations between unique subjects, thereby indirectly calculating recommendations or projections of relevant research content for users. The aim is to ensure that the suggested research content is relevant to the researcher’s work.

### Research gap

The commonly used methodologies in recommender systems are analyzed to determine if the issues mentioned with the recommender system can be resolved. Genuinely enabling the identification of RS capabilities in settings that exhibit a never-ending stream of services is crucial. A resource provisioning method is required to solve the issues raised, and provide a better suggestion to the research user. Some of the major research gap identified in the domain of recommending research article are:

1. How can one provide high Quality user satisfying Suggestions?
2. How can the suggestions by the recommender system be relevant even if the information is sparse?
3. Rapid assessment of the large-dimensioned users’ ratings.

### Objectives of the research

The primary aim of this paper includes

- To compare current traditional similarity measure methods and to improve them based on the ACBS method.
- To implement an algorithm that focuses on two weighing factors
  ○ **User Confidence**—Users are confident based on dedicated time and effort they invest in their research
  ○ **Context of Time**—Demonstrates the critical relationships between rating time and rating accuracy
- To evaluate the effectiveness of the User_UsCoTc by conducting a comprehensive comparison of popular CFL techniques against the proposed method.

The article continues with a structured presentation of the following: A review of related work in similarity measurements is described in Section 2. Section 3 introduces the proposed algorithm’s main concept. In Section 4, we use an experimental evaluation on the dataset to verify the effectiveness of the suggested strategy. Section 5 presents the conclusion of the study and offers recommendations for future work.

## Related work

The most commonly used methods in the field of recommender systems are user-based as well as item-based CFL methods. These algorithms are popular because of their clarity, simplicity and acceptable level of accuracy, and they are widely used in both industry and academia. However, some issues still need to be resolved, such as the cold problem, sparsity of data issue, and the need for high-quality recommendations. As a result, several researchers have explored ways to address these challenges. One proposed solution to mitigate the effects of data sparsity [11] is the development of a new effective CFL technique that is based on the clustering of user preferences, which aims to mitigate the effects of data sparsity. However, a potential limitation of collaborative recommender systems is their openness, which makes it difficult to prevent malicious users from entering bogus profile data. The author in this [12] incorporated social trust of users into the recommender system and developed a belief system between them. A CFL technique based on dual clustering and user belief was proposed to handle data sparsity and cold start problems [13]. The authors suggested a strategy based on different alternative viewpoints of reliability metrics in this paper [14] to increase the data sparsity issue. Table 1 shows a summary of several prominent models that have focused on different target objects. Such a highlight delineates the breakthrough of CFL approaches in its initial research period.

**Table 1:** Breakthroughs for certain CFL approaches and their focus.

Research Year	Reference	Name of the Model	Focus
1992	[15]	Tapesty	Users
1995	[16]	Bellcore Video	Movies
1997	[17]	Fab	Websites
2000	[18]	NewsDude	News
2002	[19]	NutKing	Tourism
2006	[20]	Flim Trust	Movies

With the emergence of the World Wide Web and big data [21], recommendation systems are becoming increasingly prevalent. The goal of recommendation systems is to provide multiple services to various users. To make predictions and recommendations, CFL based recommendation systems utilize information from an active user’s neighborhood. The process of CFL recommendation is typically classified into four parts.

To begin, a user-item rating matrix is built by collecting user ratings following browsing or purchasing behavior, then cleaning, converting, and inputting the data to create a matrix of user-item ratings. Second, similarity computation: CBS, ACBS, EDBS, and other ways of CFL techniques are widely used to compute similarity. The similarity between each user is then sorted with other users. Thirdly, neighborhood selection: the ideal KNN (K-Nearest Neighbors) is chosen to form the anticipated set or establish the similarity or resemblance threshold. Based on the result of the user similarity ranking, those users whose similarity exceeds the threshold values are selected as the target user’s neighbors. Finally, after collecting the target user’s nearest neighbor set, Top-N list of recommendations is presented to the user.

The most significant aspect of the CFL recommendation algorithm is the calculation of user similarity. Many user similarity metrics have been suggested for computing the similarity between two users using the matrix of user-item ratings. Among these, the CBS is the most widely used similarity measures in CFL recommendation algorithms [22]. Similarities across multidimensional facets can be compared using common similarity assessments. In user-based CF techniques, authorized members are given suggestions based on highly rated popular items by other users. On the other hand, the item-based CF technique compares items, utilizing averaged meta critic scores, and suggests additional items that are similar to an authorized member.

Let *U* = {*u*_1_, *u*_2_,…*u*_*m*_} be a collection of users and *I* = {*i*_1_, *i*_2_,…*i*_*n*_} be a collection of items. The matrix of user_item ratings is denoted as = [*r*_*ui*_]^m*n^. Where, m represents the total number of users and n represents the total items. *r*_*ui*_ refers to the user u’s rating on item i. *r*_*u*_ represents the average user’s rating. The equation for the CBS is defined as follows (1):

$${sim(u,v)}^{COS\_user}=\frac{r1.r2}{\left|\left|r_{u}\right||.||r_{v}|\right|}=\frac{\sum_{i\in I_{uv}}r_{u_{i}}r_{v_{i}}}{\sqrt{\sum_{i\in I_{uv}}r_{u_{i}}^{2}}\sqrt{\sum_{i\in I_{uv}}r_{v_{i}}^{2}}}$$ (1)

Where *I*_*uv*_ denotes common rated projects by user *u* as well as *user v*.

The CBS, on the other hand, ignores the problem of disparate rating scales. The ACBS corrects it as shown in (2).


$${sim(u,v)}^{ACOS\_user}=\frac{\sum_{i\in I_{uv}}(r_{u_{i}}-\bar{r_{u}}).(r_{v_{i}}-\bar{r_{v}})}{\sqrt{\sum_{i}{\left(r_{u_{i}}-\bar{r_{u}}\right)}^{2}}\sqrt{\sum_{j}{\left(r_{v_{j}}-\bar{r_{u}}\right)}^{2}}}$$ (2)

The PECC, is a commonly used similarity metric in CFL. It calculates the linear correlation between two objects [23]. The equation for PCC can be written as (3):

$${sim(u,v)}^{PCC\_user}=\frac{\sum_{i\in I_{u}\cap I_{v}}(r_{u_{i}}-\bar{r_{u}}).(r_{v_{i}}-\bar{r_{v}})}{\sqrt{\sum_{i\in I_{u}\cap I_{v}}{\left(r_{u_{i}}-\bar{r_{u}}\right)}^{2}}\sqrt{\sum_{i\in I_{u}\cap I_{v}}{\left(r_{v_{j}}-\bar{r_{u}}\right)}^{2}}}$$ (3)

Additionally, the EDBS is widely used for similarity assessment [24]. When data is dense and continuous, the EDBS is an appropriate method to compute similarity, as described in (4):

$${sim(u,v)}^{EDS\_user}=\frac{1}{1+\sqrt{\sum_{i\in I_{uv}}{\left(r_{u_{i}}-r_{v_{i}}\right)}^{2}}}$$ (4)

Almost all of the similarity measurements listed above are basic and do not account for the other attributes of the users. In addition to the co-rated items, there are several other characteristics that can be considered when determining user similarity.

The proposed work presents an improved similarity measure to enhance the performance of evaluating similarity. This measure takes into account certain essential elements such as confidence of users, rating item, time context and fine tunes the parameters for better accuracy.

## Materials and methods

The standard method of searching for active user’s neighbours in CFL is based on the grading details of familiar items rated or graded by two similar users. However, the earlier similarity calculation methods have several shortcomings, such as, not considering the confidence of the and ignoring the context of time in the information rating.

The emphasis each person places on different areas in their daily life varies. Some people devote a significant amount of time and effort to a particular field, making their statements more authoritative. For instance, dancers who also act as appraisers seem to spend more time in the field than regular people in the contest, “I am a dancer” leading to their statements being seen as more authoritative. This results in each user having a unique value when computing user similarity and more confidence, especially if they are experts in the relevant field. Subsequently to increase accuracy, the ACBS approach takes into account user confidence.

**Fig 1:**
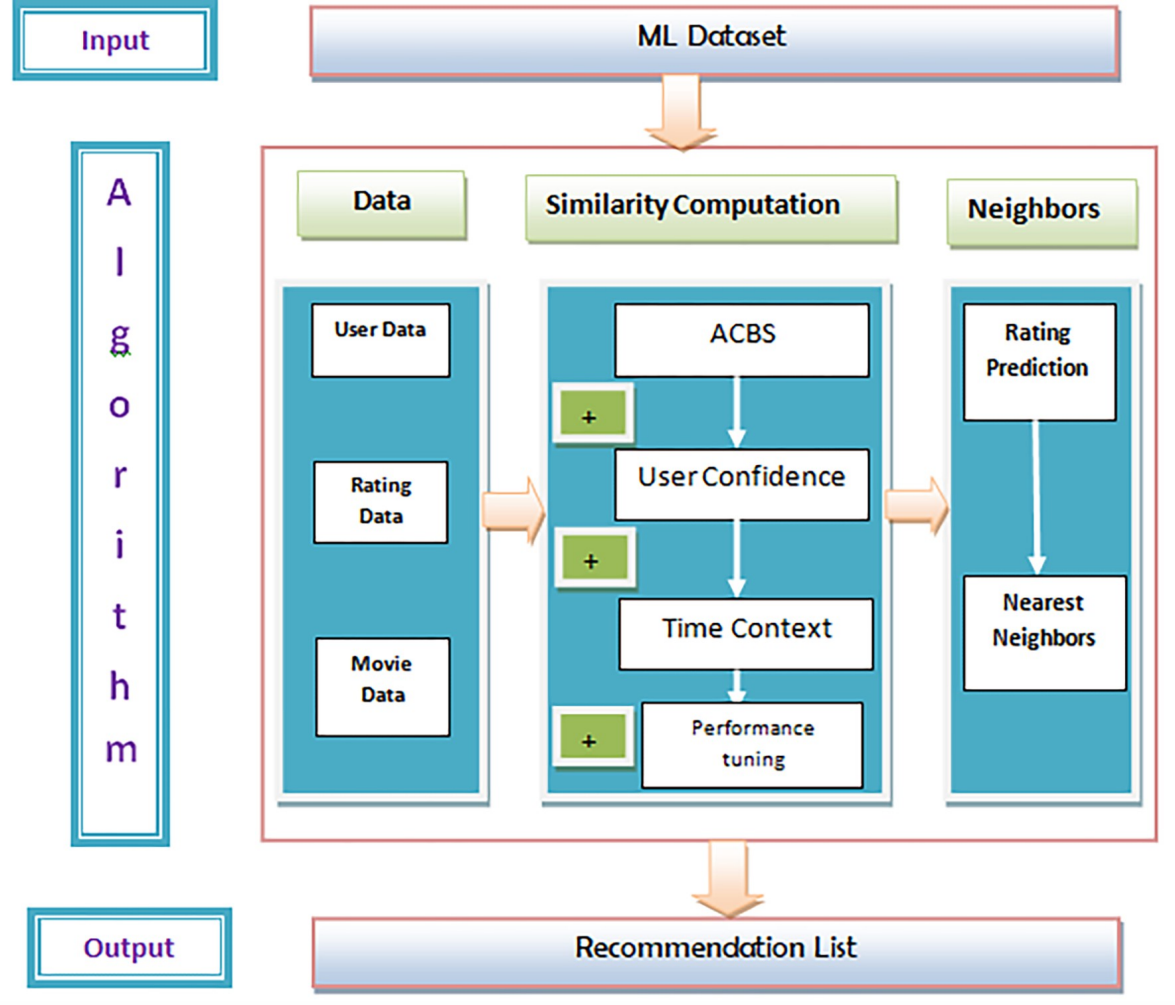
Process diagram.

This research developed a CFL based on both user confidences, time context and parameter tuning to address these disadvantages. The addition of user confidence and time context to the ACBS is described in Fig 1.

### Time attenuation

Psychologists divide human memory into two categories: (i) short-term memory and (ii) long-term memory. People can turn short-term memory into long-term memory by accumulating reviews or recalling thoughts. Furthermore, a user’s short-term interest can also be changed into a long-term one to maintain a comparatively longer period of time.

**Fig 2:**
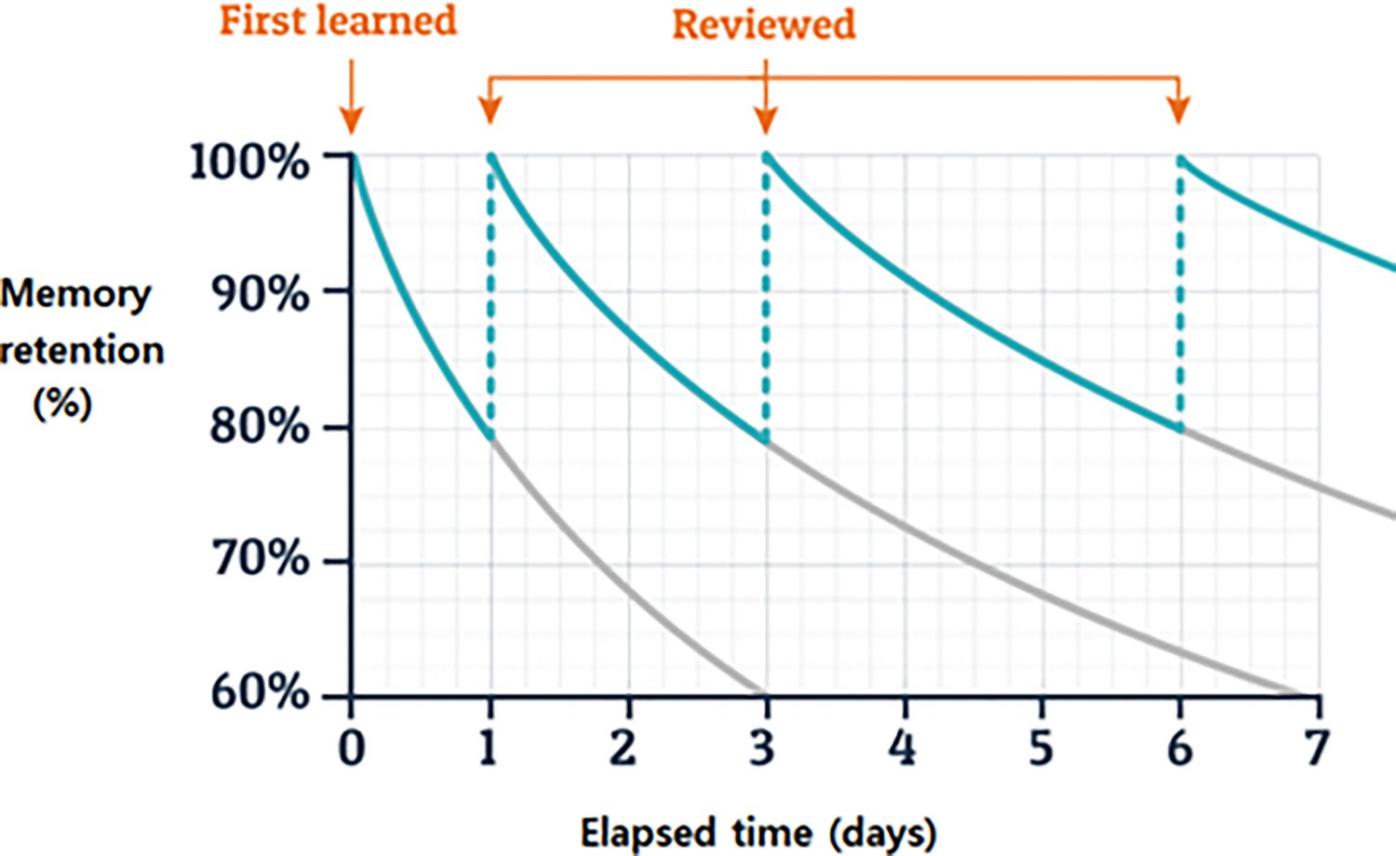
EF curve.

In the 19th century, a German psychologist Hermann Ebbinghaus, in his book "About Memory," discovered the laws of human memory forgetting. He recorded the number of items he could recall after a certain period of time and compared it to the original amount. He found that as time passed, the rate of forgetting decreased and the amount of forgetting also dropped. Based on his experimental results, he created the Ebbinghaus forgetting (EF) curve, as depicted in Fig 2, which shows the relationship between time and memory.

Contrary to popular belief, Ebbinghaus did not invent the fun slide, but instead, he uncovered how memory loss occurs over time. The graph indicates that when we first learn something, the knowledge disappears at an exponential pace, meaning we lose most of it in the first few days before the rate of loss slows down. Ebbinghaus identified the elements that contribute to memory loss when he discovered the exponential decline of memory. He also found that it is easier to remember information that is built upon prior knowledge, and the rate of decline decreases with each repetition of training. The testing effect also supports this, as testing a person’s memory strengthens recall. Repeated training interventions, as part of a learning campaign, help to consolidate the material by promoting active memory.

The following Eq (5) is an exponential time decay function for time-weighted user interest proposed in the literature [25]:

$$\omega (t,t(R_{u_{i}}))=e^{-\lambda (t-t(R_{u_{i}}))}$$ (5)

Where λ is weight coefficient, t denotes current behavior of object time, and $t(R_{u_{i}})$ represents the end time that the user took action on project.

### User confidence

The use of CFL based on neighbours is common. The similarity formula is widely used to determine the similarity between two users. Traditional methods of calculating similarity between two users are not feasible due to the presence of sparsity in the rating matrix [26]. For instance, calculating the formula using existing similarity approaches may result in a high similarity if two users have only one shared rating item. Incorporating item popularity in evaluating user interest similarity can enhance recommendation quality [27]. However, if both users purchase the same book, “Artificial Intelligence Introduction,” their interests are likely to be similar in nature, as only someone interested in data mining would purchase it. As a result, if users take the same action on uncommon items, the commonality of their attentiveness can be more clearly demonstrated. The ACBS numerator now, is changed to a new equation, which is defined as shown in Eq (6):

$$\sum_{i\in I_{u}\cap I_{v}}((r_{u_{i}}-\bar{r_{u}})(r_{v_{i}}-\bar{r_{v}})+\frac{1}{log(1+|N(i)|)})$$ (6)

Where *N*(*i*) denotes the No of rating item i.

Professionals who invest more time and energy in their respective fields have a deeper understanding of their area compared to the average person. Hence, their knowledge of current events in their field is higher. From this standpoint, we agree that those who will spend more time and energy in their respective fields are more credible than others. As a result, we can multiply the outcome of the similarity computation of users by user confidence, to arrive at a more convincing conclusion. The equation for this is defined as shown in Eq (7).


$$\emptyset \;{\left(z\right)}^{Area\_D}=\frac{1}{1+e^{-(number(z)-ave)}}$$ (7)

Where *number*(*z*) denotes number of rating movies by user ‘z’ and ‘ave’ denotes average rating items by users.

The interest of user has age stratification characteristics, and users of different ages have varied interest hierarchies. To differentiate between different ages, the user’s age is divided into nine tiers, as shown in Table 2. To compute the age confidence between two users, we use the following Eq (8).


$$\emptyset \;{\left(a,b\right)}^{Age\_D}=1-\frac{\left|{Age}_{a}-{Age}_{b}\right|}{8}$$ (8)

Where *Age*_*a*_ denotes age fragmentation of user a and *Age*_*b*_ denotes age fragmentation of user b.

**Table 2:** Segmentation of user age.

Age	Segmentation
1–10	1
11–20	2
21–30	3
31–40	4
41–50	5
51–60	6
61–70	7
71–80	8
>80	9

The following Eq (9) is used to compute the overall confidence of user in the field, as well as age confidence.


$$\emptyset (a,b)=\emptyset {\left(a,b\right)}^{Age\_D}+\emptyset {\left(a,b\right)}^{Area\_D}$$ (9)

As a result, we improvise ACBS by including user confidence in Eq (6), as shown below.


$${sim(u,v)}^{UD\_user}=\frac{\sum_{i\in I_{u}\cap I_{v}}(r_{u_{i}}-\bar{r_{u}})(r_{v_{i}}-\bar{r_{v}})+(1/{log}_{2}(1+N(i)|)))}{\sqrt{\sum_{i}{\left(r_{u_{i}}-\bar{r_{u}}\right)}^{2}}\sqrt{\sum_{j}{\left(r_{v_{j}}-\bar{r_{u}}\right)}^{2}}}\emptyset (v)$$ (10)

### Time context

People’s interests tend to change over time. For instance, young people might initially enjoy individuality in their hair color and clothing style, and as time passes, but as they grow older, their fashion choices tend to shift towards a more mature look. Similarly, modern engineers may start by purchasing books with classic examples, but as they gain more experience, they tend to opt for instructional books that are more in-depth. A latest thing may catch people’s attentiveness at first, but after some time has passed, it may be forgotten. A latest trend may initially attract people’s attention, but it may eventually be forgotten as time passes. The lifespan of different products also varies, with news typically having a short lifespan, while dramas may last for a longer period. Similarly, movies have a shorter lifespan and may eventually lose their appeal after a certain amount of time [28].

If the convergence among both users’ items has a substantial fraction of intersection, the great similarity between the two can be conveyed to some extent [29]. The equation based on the perspective is as shown in (11).


$${sim(u,v)}^{TC\_User}=\frac{\sum_{i\in I_{u}\cap I_{v}}(r_{v_{i}}-\bar{r_{v}}){\left(r_{v_{i}}-\bar{r_{v}}\right)}^{e^{-\mu |t_{u_{i}}-t_{v_{i}}|}}}{\sqrt{N(v)N(u)}}$$ (11)

Where $t_{u_{i}}$ and $t_{v_{i}}$ are information of time of u and v users when the users rated the item I respectively. *μ* denotes degree of interest change rate and this proper value can be obtained from repetitive experiments.

### Proposed technique

Based on confidence of user and time context, this study provides a CFL recommendation system. The fundamental idea is to improve the ACBS by adding user confidence as well as time context to it, tuning the parameters and then compute the similarity between users [30]. The improved ACBS equation represented as shown in (12).


$${sim(u,v)}^{User\_UsCoTc}=\alpha .{\beta .sim(u,v)}^{UD\_User}+(1-\beta ).(1-\alpha ).{sim(u,v)}^{TC\_User}$$ (12)

Where α and β denote impact factors that are being adjusted to obtain the optimal results by repetitive experiments. The impact factors indicate the correlative bounds among various users with respect to user confidence and time context.

#### Prediction value

To compute the forecasting for an unrated item I by user u, obtain the user u’s set k neighbours. We can calculate the similarity values among user u and with other users using Eq (12). The neighbor set is then built based on the user similarity measures by picking the first L users who are closest to user u. We may also calculate the prediction value using the following Eq (13):

$${P_{u_{i}}}^{User}=\bar{R_{u}}+\frac{\sum_{v\in L}sim(u,v).(r_{v_{i}}-\bar{r_{v}})}{\sum_{v\in L}\left|sim(u,v\right)|}+(\alpha +\beta )$$ (13)

Where $P_{u_{i}}$ denotes active user u’s precision on I, which is the target item, L is the neighbor set of u.

We present our proposed methodology as shown below with a clear description:

**Algorithm 1. Step by Step procedure of the methodology**.

*Input required*: *R is the rating matrix; μ and α are the threshold values*.

*Output expected* $P_{u_{i}}$*, prediction of u, the active user*

*Step_1: To compute user similarity, apply Eq (12), and the resulting similarity matrix is referred by*

*sim(u*, *v)*^User_UsCoTc^

*Step_2*: *In descending order*, *sort the similarity matrix sim(u*, *v)*^User_UsCoTc^

*Step_3*: *Obtain the active user u’s neighbor selection range*, *and then construct a neighbor set that is based on user similarity*. *Impact factors* α *and* β *are being adjusted to* obtain the optimal results.

*Step_4: To predict the rating $P_{u_{i}}$, utilize Eq (13) with parameters tuning of* α *and* β

### Experiments and results

In this study, we incorporated GroupLens datasets [31] which are publicly available for experimental evaluations. GroupLens develops social/virtual communication theory and practice by creating and comprehending platforms utilized by actual users. The GroupLens program comprises of its experimental recommender systems (not only for MovieLens and Usenet News; but also, for the most generalized domains) and the outcomes of several months of commercialized recommender implementations. To help societies of CFL system participants, it emphasizes significant research findings and trials with innovative and varied layouts.

**Fig 3:**
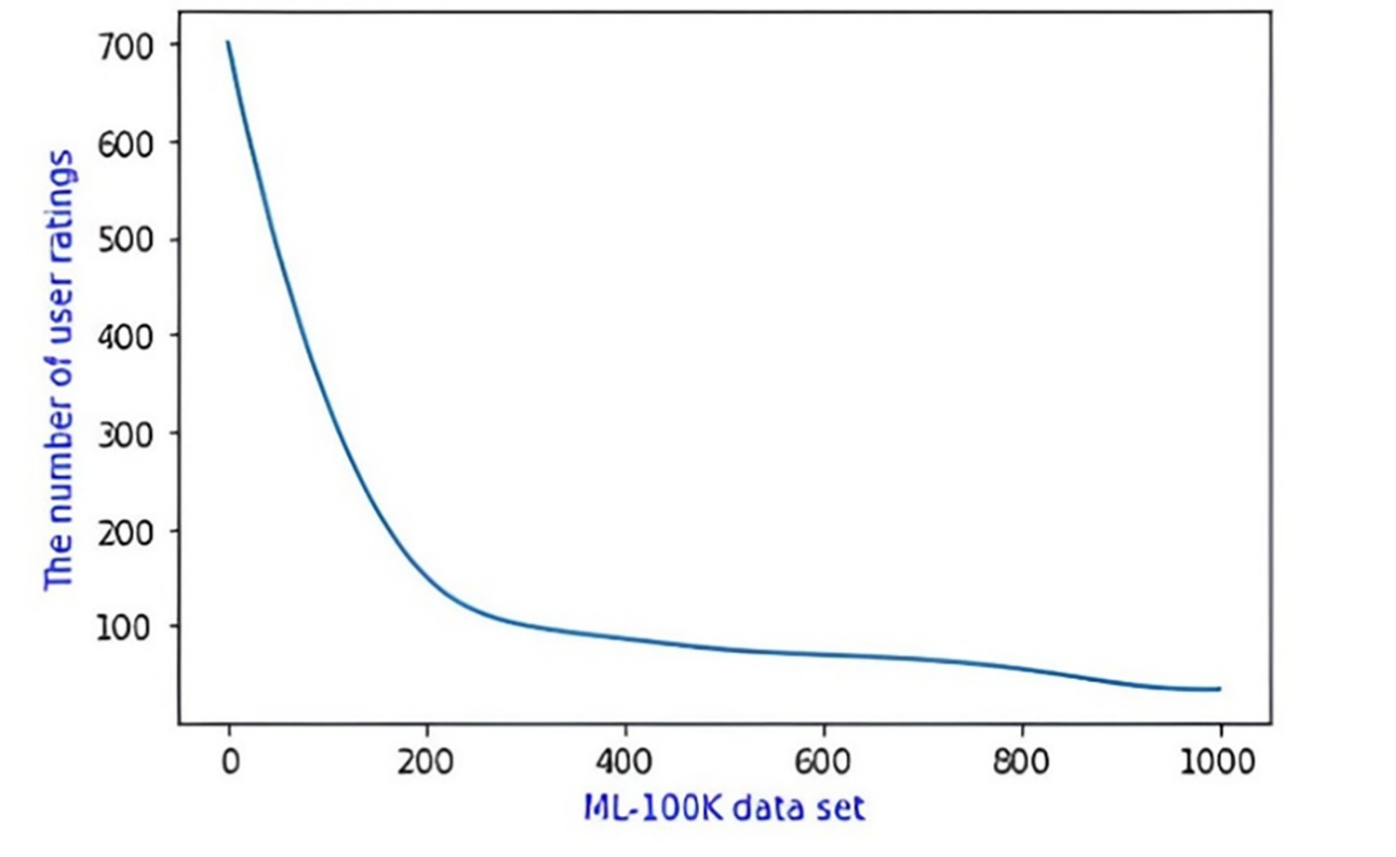
ML-100K data set.

GroupLens Research donated the data set, which is a film rating data set. The data set contains 100,000 ratings for 1682 films from 943 individuals, with each user rating at least 20 films and a rating interval of 1–5, as shown in Table 2. Meanwhile, the data set has a sparseness of 1−100000/(943*1682) = 93.7%. The number of items rated by numerous users is less than 100, as shown in the table. In descending order, the items are rated by users on the dataset as is shown in Fig 3. As depicted, it is clear that the number of items that are rated by users is less than 100.

The data set is classified into two parts to assess the algorithm’s performance: 80% of the data set is used as the training set, and 20% is used as the test set. There are four attributes concerning users’ attribute features in the ML-100k data set: gender, occupation, age and zip code. The matrix of user item rating is shown in Table 3.

**Table 3:** User-item rating matrix.

	Movielens-100k
Users	943
Items	1682
Ratings	100000
Rating Scale	{1,2,3,4,5}
Sparseness of Data	93.7%

#### Evaluation

The basic measures for measuring the quality of a recommendation system are MAE i.e., mean absolute error and RMSE i.e., root mean square error [32, 33]. The average absolute difference among forecast values as well as actual ratings is defined as MAE, an extensively used measures for evaluating recommendation accuracy [34].

Assume the set of user predicted rating is {*y*_1_, *y*_2_, *y*_3_……*y*_*n*_) and collection of ratings that are actual is {*x*_1_, *x*_2_, *x*_3_……*x*_*n*_), the MAE is computed as stated in (14).


$$MAE=\frac{\sum_{i=1}^{n}\left|y_{i}-x_{i}\right|}{n}$$ (14)

Where n represents the number of rating movies.

The RMSE equation is given by (15).


$$RMSE=\sqrt{\frac{\sum_{i=1}^{n}{\left|y_{i}-x_{i}\right|}^{2}}{n}}$$ (15)

The more accurate the forecasts are, the lower the MAE and RMSE will be. Precision, F1 measure and recall are frequently used to assess the correctness of top-N recommendations.

The recommendation items are S(u), and the test actual items are A(u). The following Eq (16) is used for precision.


$$precision=\frac{\sum_{u}\left|S(u)\cap A(u)\right|}{\sum_{u}\left|S(u)\right|}$$ (16)

The metric recall is depicted as shown in (17).


$$recall=\frac{\sum_{u}\left|S(u)\cap A(u)\right|}{\sum_{u}\left|A(u)\right|}$$ (17)

The F1 measure is given by the Eq (18).


$$F1=\frac{2*precision*recall}{precesion+recall}$$ (18)

## Results and discussions

Experiments are carried out on the specified dataset in this part. We use metrics to compare our suggested algorithm to other algorithms. The number of chosen neighbours that are nearest really has a significant influence on the suggestions quality. As a result, the amount of nearest neighbours k varies from ten to sixty in tests. CBS, ACBS, PECC, and EDBS are now the most often used methods for calculating similarity between uses. The MAE as well as RMSE values are recorded in the table as the value of neighbors increases from 10 to 60 as shown in Tables 4 and 5.

**Table 4:** MAE values of different similarity methods as value of neighbors increases.

Neighbors →	10	15	20	25	30	35	40	45	50	55	60
EDBS_User	0.810	0.790	0.788	0.786	0.785	0.7824	0.783	0.783	0.782	0.781	0.780
PECC_User	0.790	0.778	0.776	0.775	0.776	0.775	0.776	0.774	0.772	0.770	0.766
CBS_User	0.770	0.760	0.749	0.750	0.749	0.748	0.746	0.744	0.741	0.39	0.737
ACBS_User	0.750	0.735	0.732	0.731	0.730	0.728	0.727	0.726	0.724	0.722	0.720

**Table 5:** RMSE values of different similarity methods as value of neighbors increases.

Neighbors →	10	15	20	25	30	35	40	45	50	55	60
EDBS_User	1.020	1.016	1.010	0.990	0.990	0.990	0.990	0.990	0.989	0.988	0.988
PECC_User	1.000	0.990	0.988	0.980	0.980	0.980	0.980	0.980	0.979	0.980	0.970
CBS_User	0.990	0.980	0.970	0.970	0.970	0.966	0.960	0.960	0.958	0.958	0.958
ACBS_User	0.965	0.950	0.940	0.940	0.938	0.936	0.930	0.928	0.929	0.928	0.929

**Fig 4:**
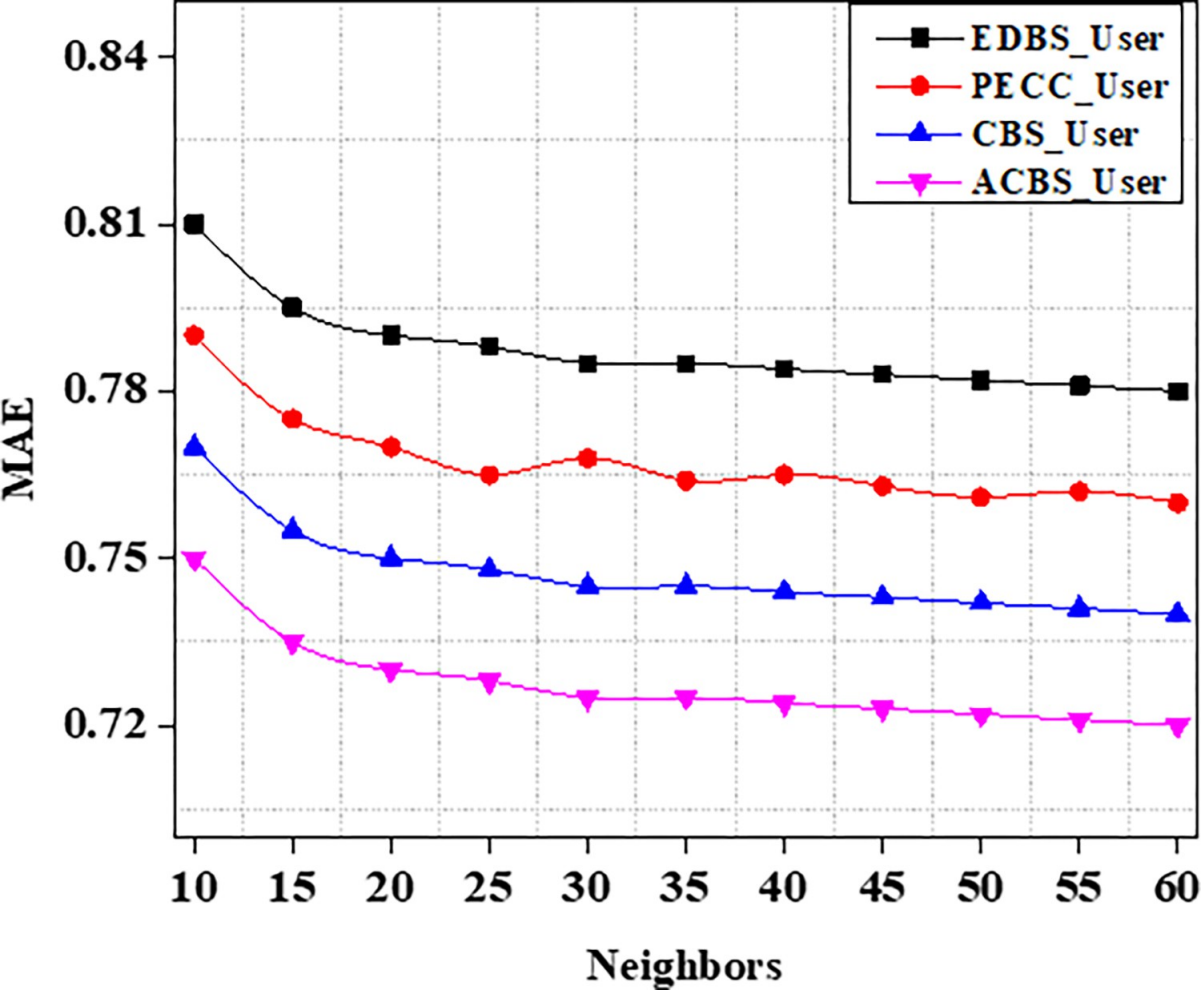
MAE.

**Fig 5:**
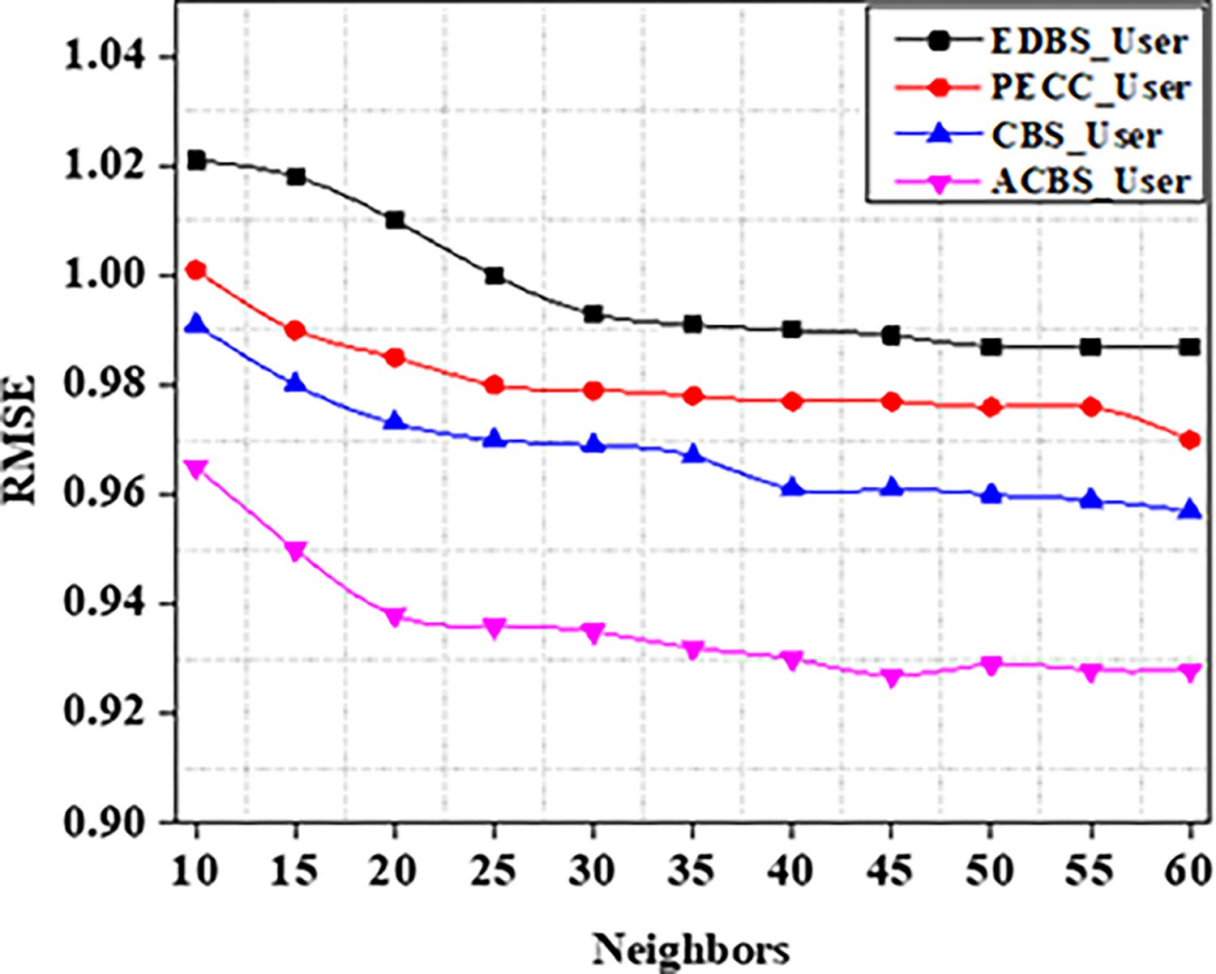
RMSE.

**Fig 6:**
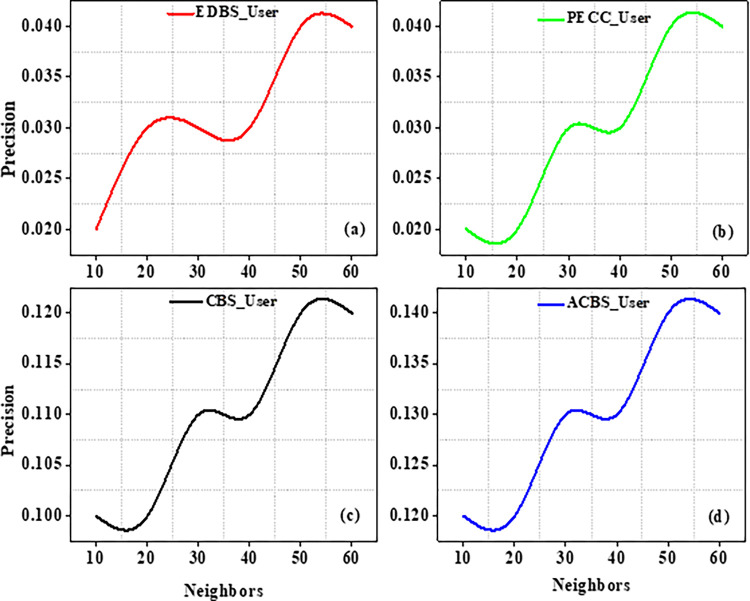
Precision with different neighbors.

**Fig 7:**
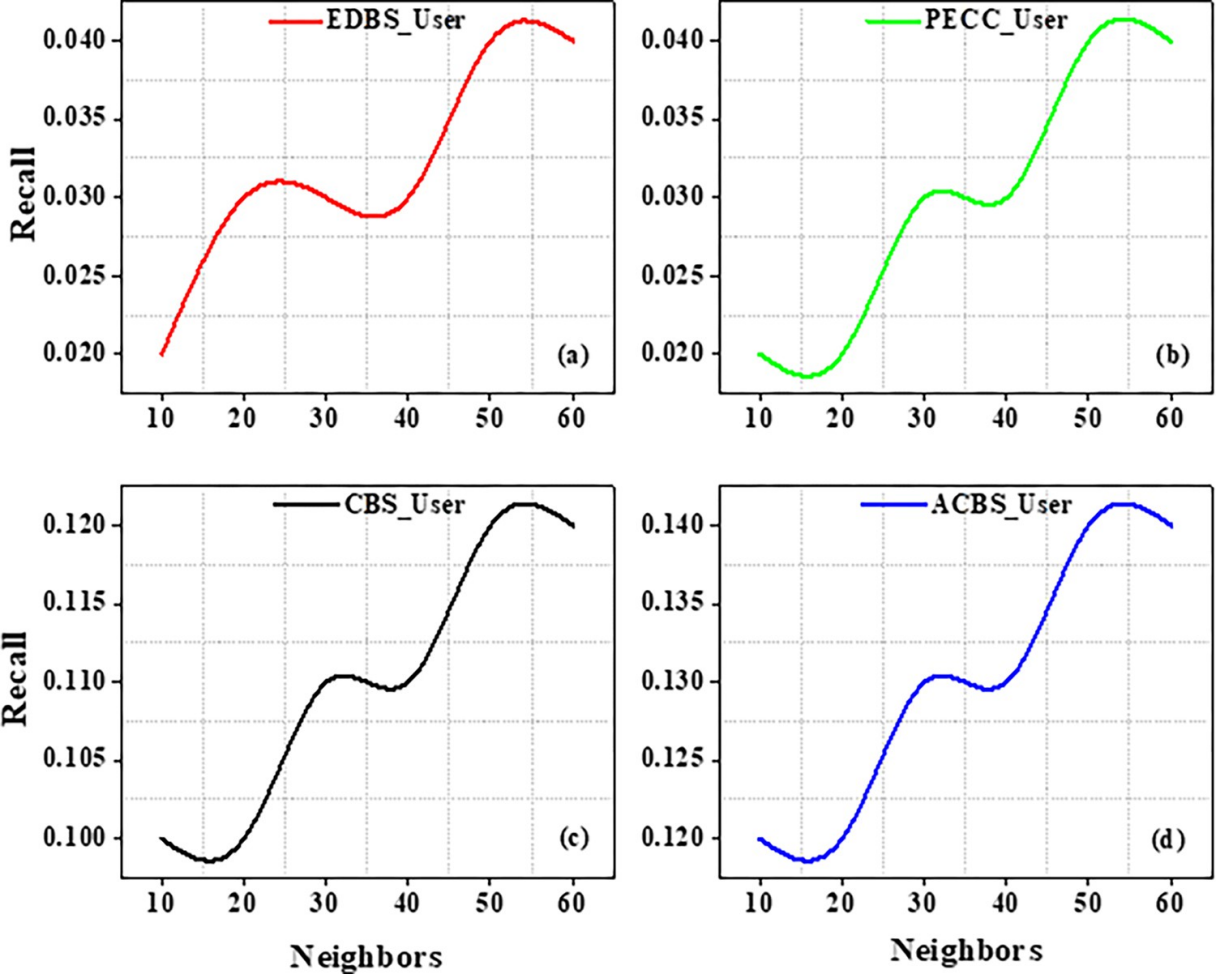
Recall with different neighbors.

**Fig 8:**
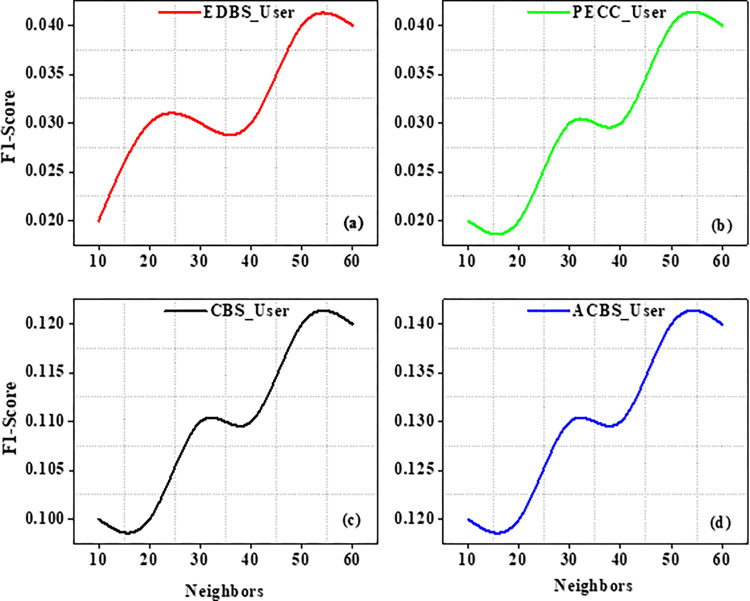
F1 scores with different neighbors.

Experiment findings on the ML 100K dataset are shown in Figs 4 and 5. It should be noticed that as the value of k increases from number ten to twenty. Furthermore, when the nearest neighbor *k* fluctuates from twenty to sixty, the EDBS, CBS, and PECC values appear to be decreasing. The ACBS values, on the other hand, decrease as the nearest neighbor k increases from twenty to thirty, and increases as the value of k increases from thirty to sixty. Some classic similarity metrics (such as CBS, PECC, and EDBS) have worse prediction accuracy than the ACBS (Figs 6–8).

**Fig 9:**
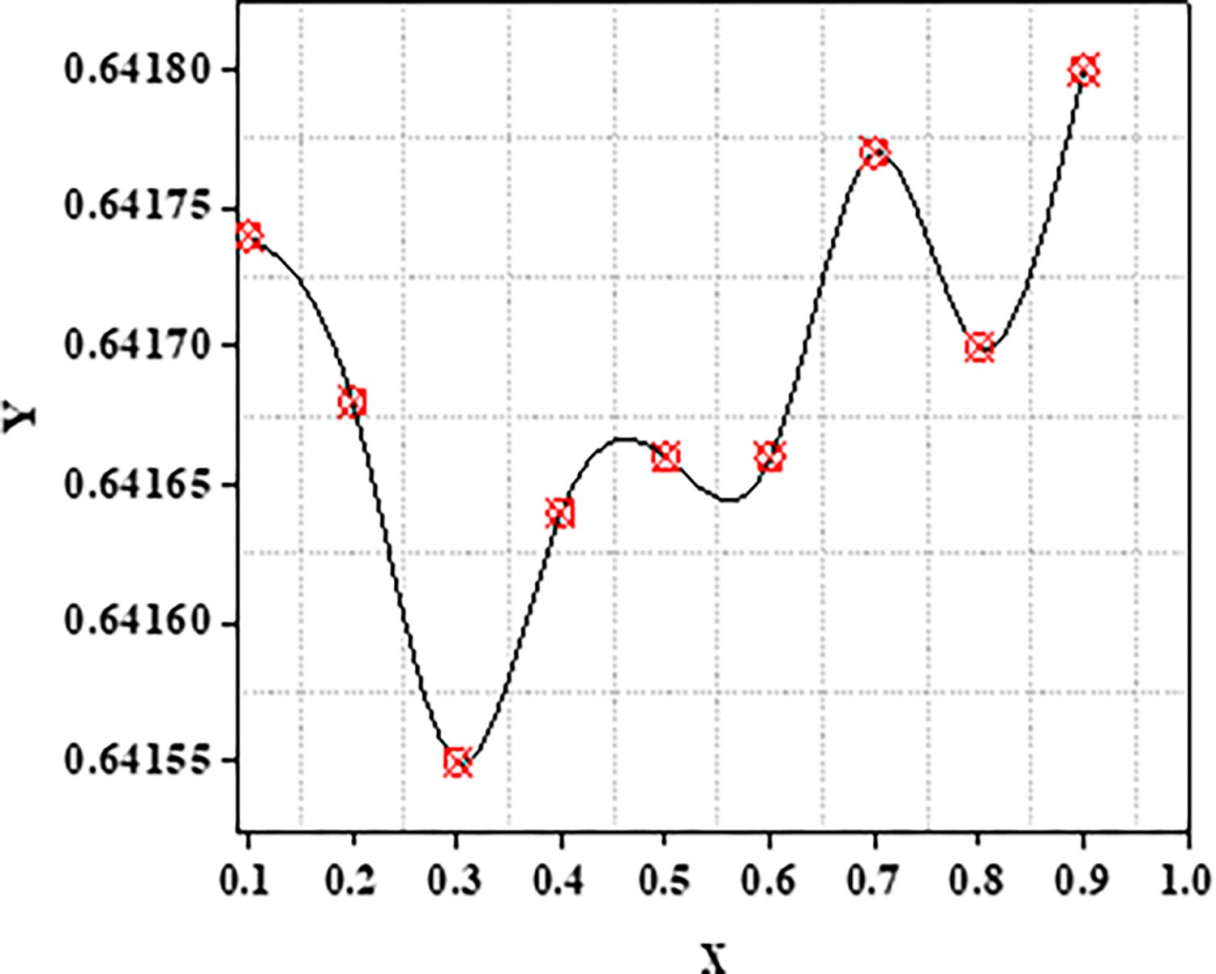
Y: MAE with various X: μ.

**Fig 10:**
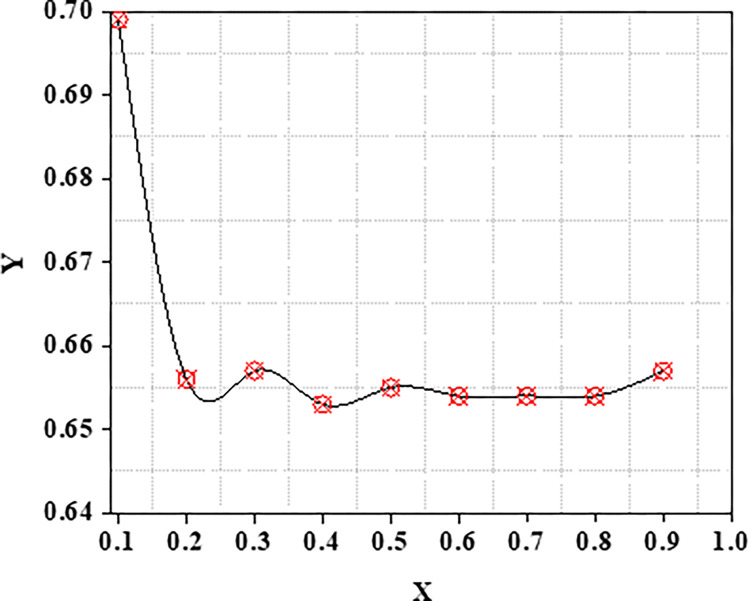
Y: Average MAE with various X: *μ*.

The metrics Recall, Precision as well as F1 scores of the standard similarity metrics are compared. The preceding testing findings have clearly shown that the ACBS similarity method clearly outperforms other methods. As a result, the ACBS in this paper’s similarity computation includes user confidence and time context. The parameter can be derived by iterating the experiments using Eq (11); we fix the nearest neighbor k to thirty. Clearly, the data suggests that the MAE is lowest when is 3*10^−8^. MAE with different μ as well as the average MAE is shown in Figs 9 and 10.

**Fig 11:**
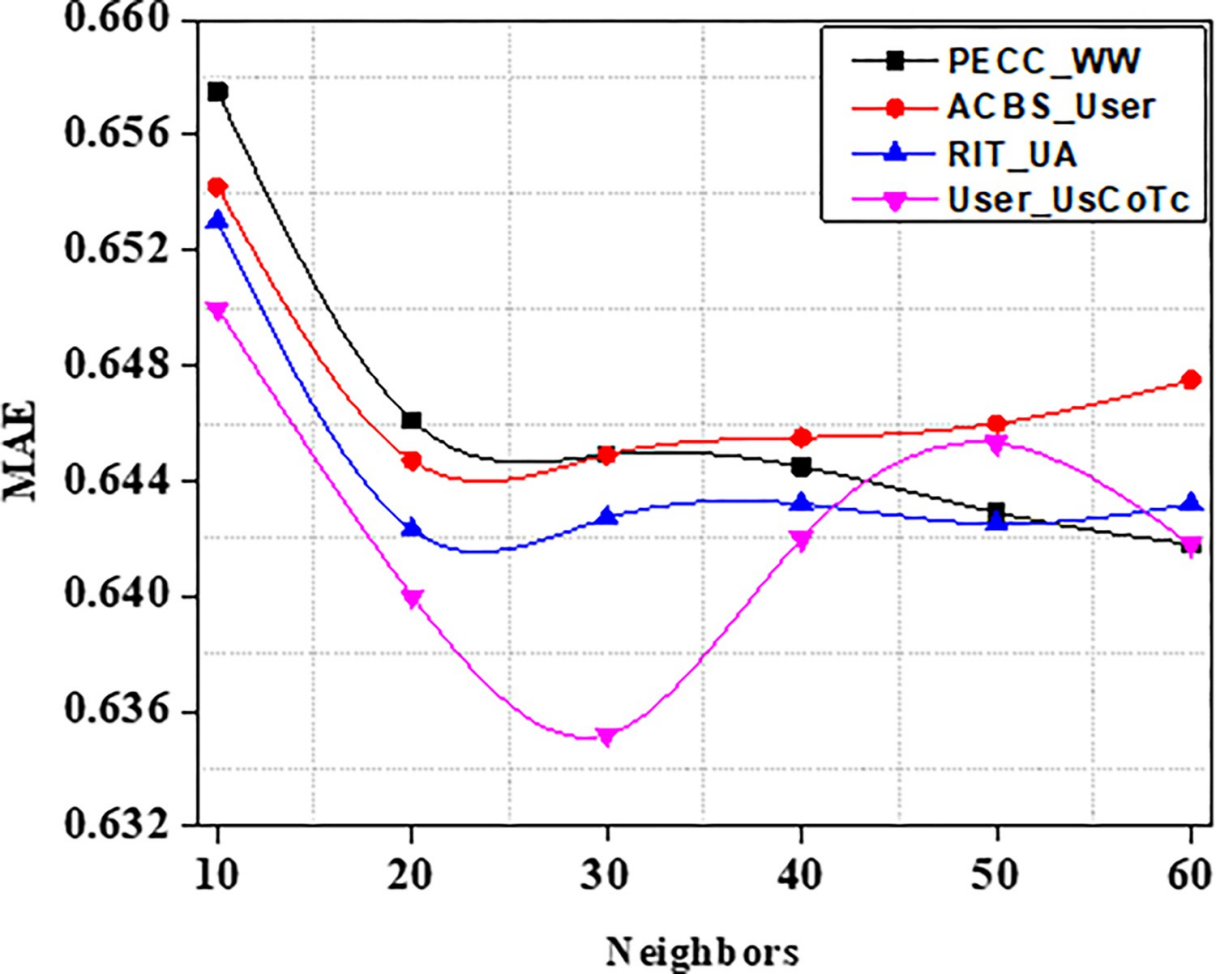
MAE with varied neighbors range.

**Fig 12:**
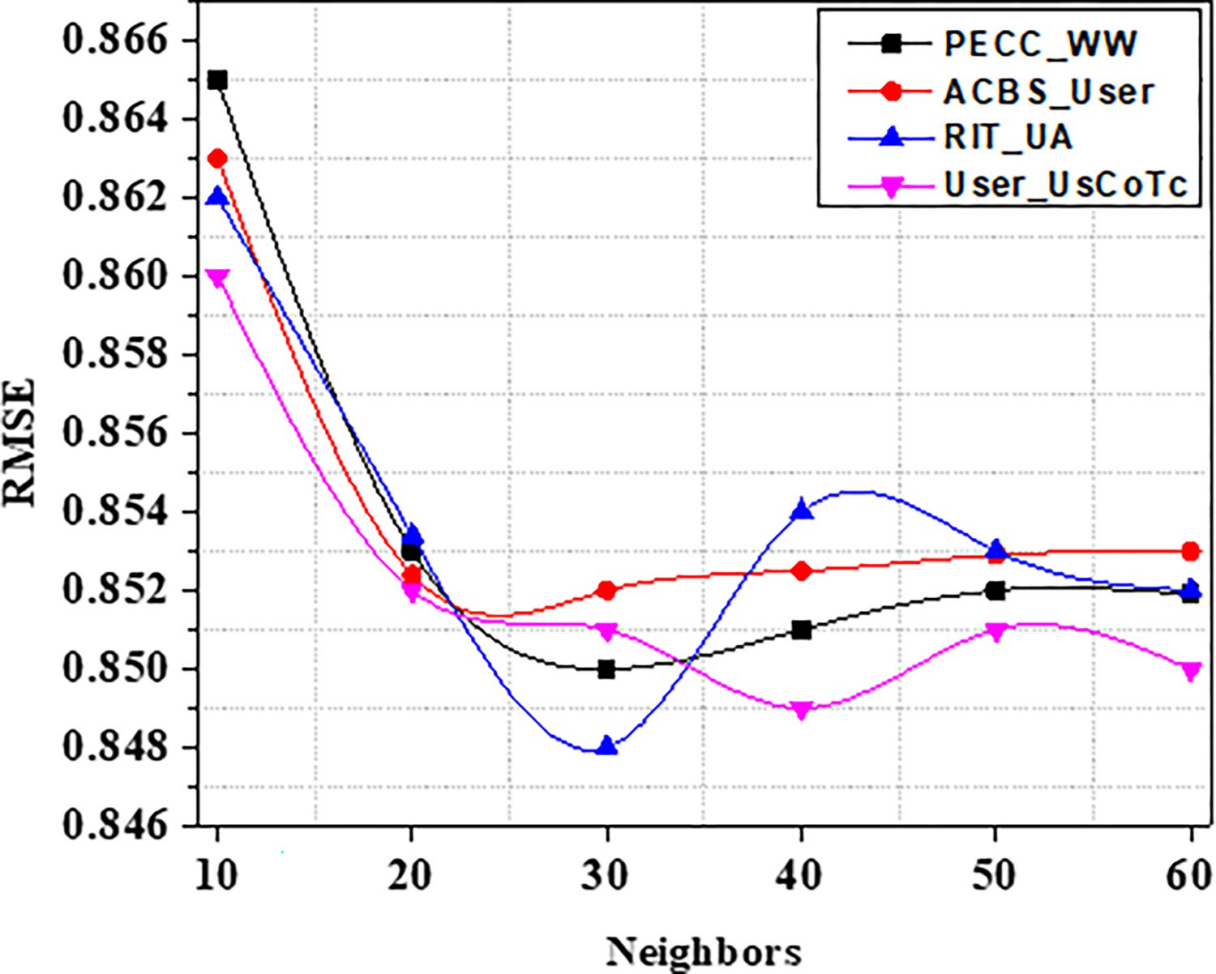
RMSE with varied neighbors range.

MAE and RMSE values of different models are recorded in Tables 6 and 7. On the ML 100K dataset, our strategy User_UsCoTc achieves the lowest MAE and also, least RMSE values when compared to various number of neighbours, as shown in Figs 11 and 12. The best recommendation quality is achieved when the number of neighbors is 25.

**Table 6:** MAE values comparison of the different models against the proposed.

Neighbors →	10	20	30	40	50	60
PECC_WW	0.6565	0.6445	0.6443	0.6442	0.6438	0.6420
ACBS_User	0.6525	0.6442	0.6444	0.6445	0.6447	0.6480
RIT_UA	0.6530	0.6420	0.6430	0.6440	0.6430	0.6440
User_UsCoTo	0.6500	0.6400	0.6350	0.6420	0.6450	0.6420

**Table 7:** RMSE values comparison of the different models against the proposed.

Neighbors →	10	20	30	40	50	60
PECC_WW	0.8645	0.8523	0.8500	0.8501	0.8520	0.8520
ACBS_User	0.8623	0.8521	0.8521	0.8523	0.8525	0.8520
RIT_UA	0.8620	0.8523	0.8480	0.8530	0.8528	0.8520
User_UsCoTo	0.8600	0.8520	0.8510	0.8490	0.8510	0.8500

The Figs 11 and 12 show that on the ML 100K dataset, our technique User_UsCoTc gets the lowest MAE and RMSE with various neighbors. The best recommendation quality is achieved with 25 neighbors, according to the experiments. The results of the experiments on the dataset support that User_UsCoTc outperforms ACBSS.

**Fig 13:**
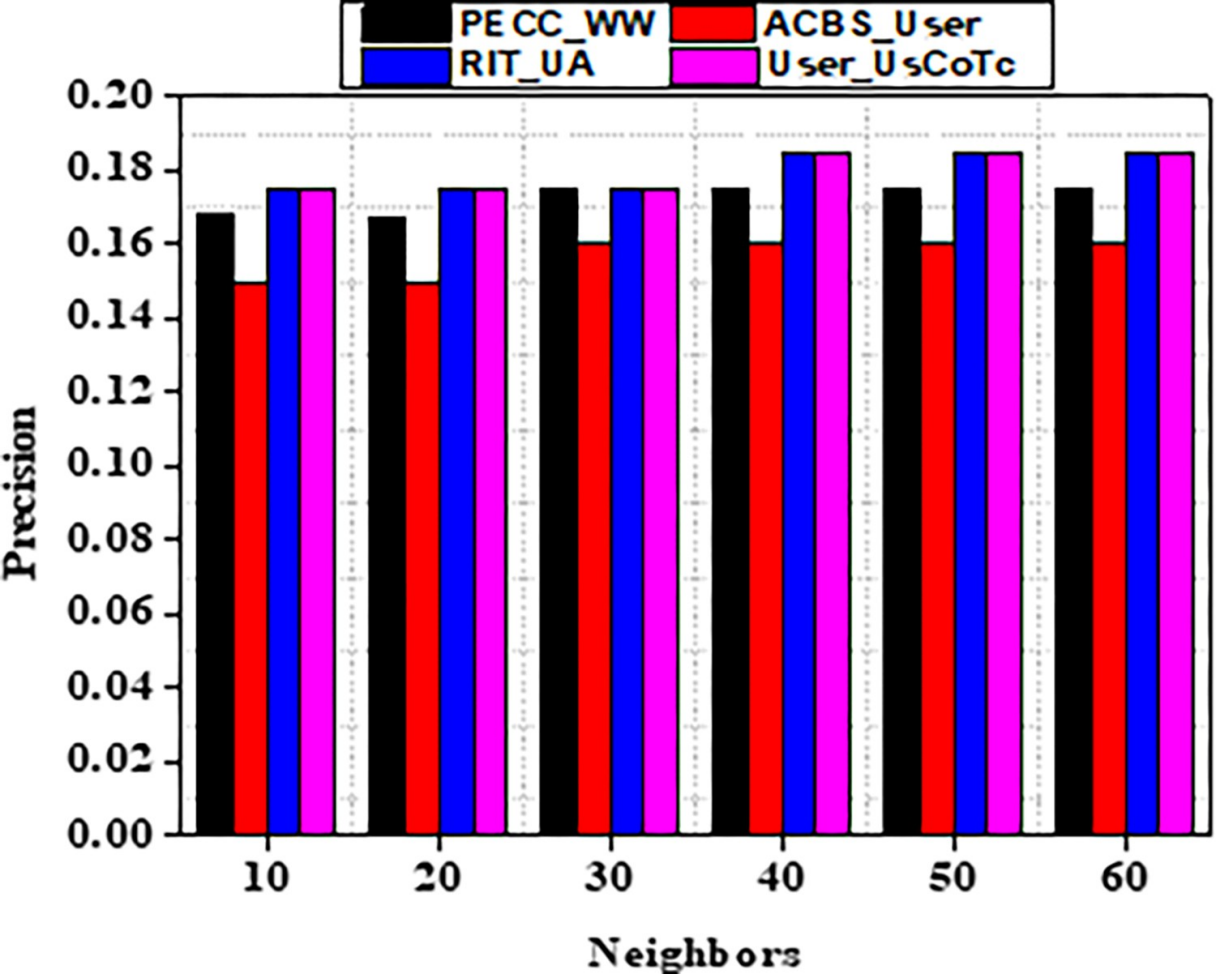
Precision with various neighbours.

**Fig 14:**
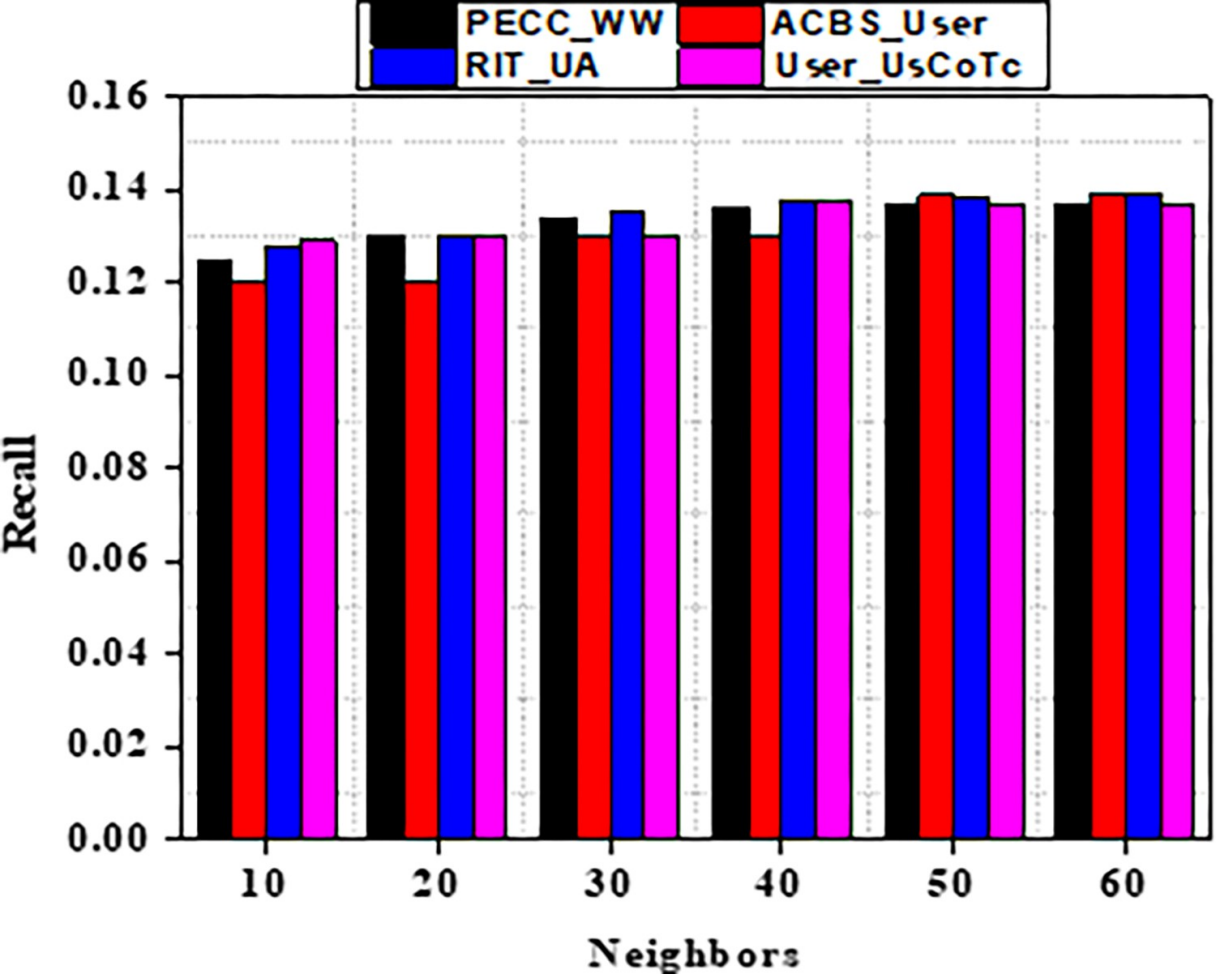
Recall with various neighbours.

**Fig 15:**
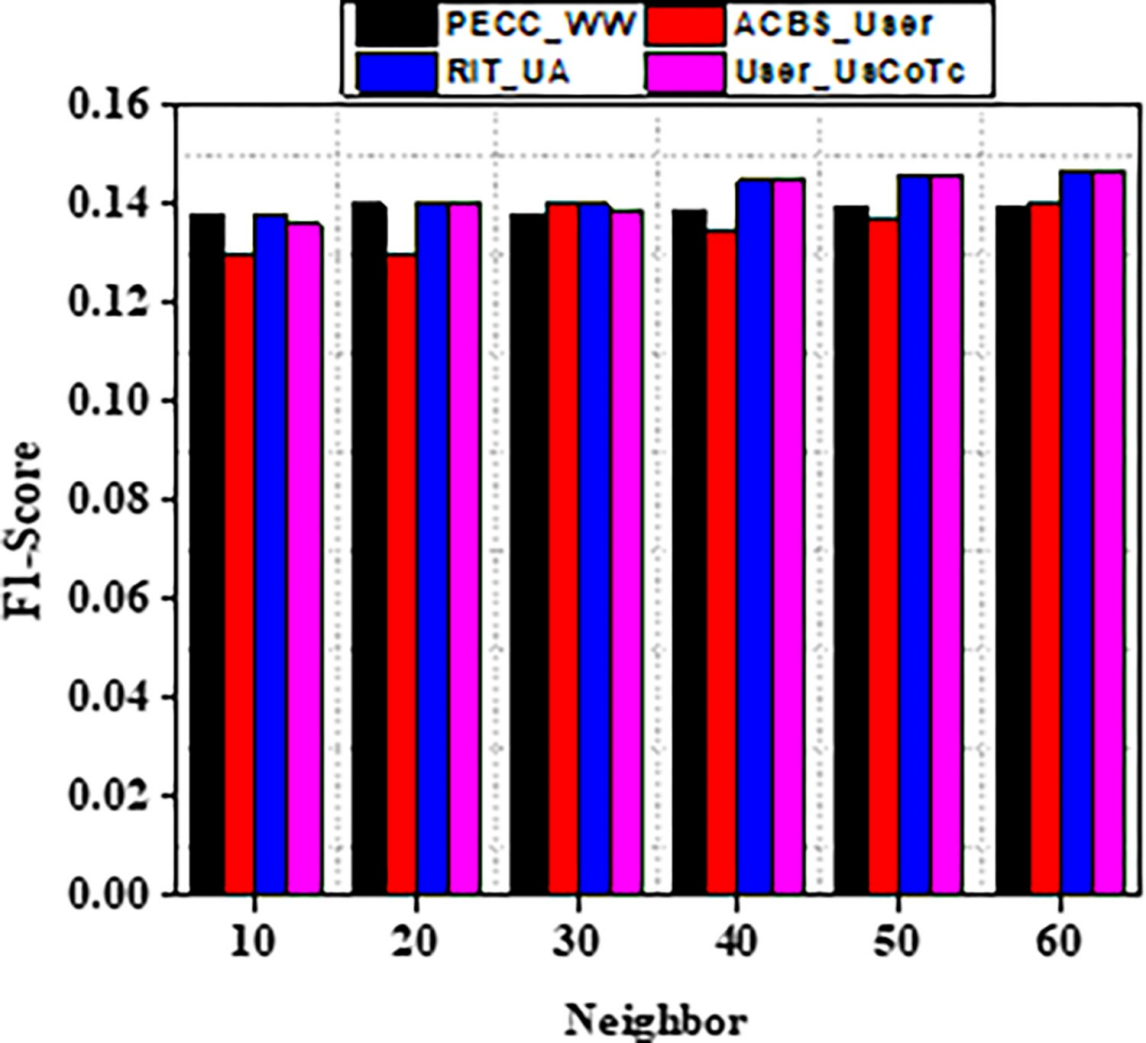
F1-measure with various neighbours.

As a result of the experiments on the given dataset, it can be concluded that the method User_UsCoTc outperforms ACBS. The comparison of User_UsCoTc with PECC_WW, ACBS_User and RIT_UA [35, 36] is illustrated in Figs 13–15.

To calculate the similarity between user u and user v in PECC_WW,

- First the local similarity, global similarity and Hellinger distance are computed.
- Secondly, the ratio of weight coefficient to weight of the local neighbors is computed [13–15].
- Finally, PECC_WW offers a prediction for recommendation.

The recommendation system displays a value between -1 and +1, where -1 indicates low correlation and +1 indicates high correlation. It is sometimes referred to as zero-order correlation because a value of 0 signifies no relationship. The main drawback of this method is that it doesn’t account for inaccuracies in similarity computation when using web services. Additionally, the output may not be reliable in situations like users only providing feedback on one variable or two users having the same rating.

Although ACBS_User improves cosine similarity performance, it lacks consideration of user rating preferences. The ACBS measure is a superior version of the vector-based similarity where different users have different ratings, i.e., some users may rate the items higher while others may rate them lower. To address the limitations of vector-based similarity, the user average rating for each user is deducted from each user’s rating for the pair of items being referred to [13–15].

The RIT-UA algorithm was developed for handling sparse data and incorporates factors such as user attribute characteristics and time decay of ratings that impact user rating behavior. This algorithm was built on the foundation of the traditional similarity calculation. The RIT-UA algorithm consists of two parts: the similarities of user rating-interest, which takes into account the similarities of user rating and interest, as well as how these change and are impacted by rating time and the confidence coefficient between users; the second part is the similarities of user attributes, which considers the impact of the user attribute feature on the recommendation and calculates the similarity of the user attributes by determining the weight of each attribute feature. The RIT-UA algorithm ultimately combines the two parts linearly [13–15].

The proposed computation approach, the User_UsCoTc shows significant improvements in all performance metrics. The accuracy rate improves by 16.2% when the number of neighbours is increased to 30. The proposed approach has a slightly higher accuracy than PECC_WW and RIT_UA. During the process of increasing the number of neighbors from 20 to 30, the experimental results reveal that the proposed method has a higher recall rate compared to other techniques. However, when the number of neighbors is increased from 50 to 60, the proposed method has a lower recall rate than RIT_UA, but a higher recall rate compared to the remaining two algorithms. Therefore, incorporating user confidence and time context using the ACBS similarity approach improves accuracy, recall, and the value of suggestions. Thus, Table 8 represents comparative analysis outcomes of various methods against three significant metrics.

**Table 8:** Comparison of various methods using vital metrics.

	EDBS_User	PECC_User	CBS_User	ACBS_User
MAE	1.44	1.41	1.37	1.33
RMSE	1.00	0.98	0.97	0.93
F1-score	0.03	0.03	0.11	0.13

The overall analysis demonstrates that User_UsCoTc increases the recommendation quality and has superior results, based on the findings and extensive analysis of the aforementioned tests. This supports the validity and feasibility of the improved algorithm presented in this paper.

## Conclusions

In this study, Collaborative filtering (CFL) method that takes into account both user confidence and time context is proposed, with optimization leading to improved performance. Our strategy is founded on the assumption that specialists in each area are more persuasive, and that users’ interests change over time. The objective of this study is to find a better metric to tweak in order to find the optimum typical similarity measure by examining common similarity algorithms and developing a new approach based on the ACBS. Experimental studies were conducted to demonstrate the superiority and suitability of User UsCoTc for calculating user similarity. The effectiveness of the proposed approach is evaluated using metrics such as MAE, RMSE, Accuracy, Recall, and F1-measure. The first element, user confidence, highlights the importance of users who spend more time and effort in their research being more convincing. The second component, temporal context, takes into account the crucial connection between rating time and accuracy. Results from experiments on the dataset showed that the proposed approach outperforms existing algorithms. Finally, the User_UsCoTc is effective in improving the performance of recommendation systems with an accuracy range of 16.2% in comparison to the existing models. In the future the objective is to compare our proposed model with assembling techniques that incorporate modern day variables in CFL, providing a different perspective for future researchers in similar domains.
